# Hospital Corps, 74th Regiment, N. Y. N. G.

**Published:** 1899-02

**Authors:** 


**Monthly Review of Medicine and Surgery.**

*EDITORS:*

THOMAS LOTHROP, M. D. - WM. WARREN POTTER, M. D.

All communications, whether of a literary or business nature, should be addressed to the managing editor: 284 FRANKLIN STREET, BUFFALO, N. Y.

VOL. XXXVIII. FEBRUARY, 1899. No. 7.

### HOSPITAL CORPS, 74TH REGIMENT, N. Y. N. G.

AT ONE of the last of the series of battalion drills to be given by the 74th Regiment in the old armory, held on January 5, 1899, the regiment was reviewed by Colonel Smith and other officers of the 13th Regiment, U. S. Army, now stationed at Fort Porter.

The program announced as a special feature of the evening a drill of the hospital corps by Major William G. Bissell, Surgeon of the 74th Regiment. After the reviewing of the regiment Major Bissell called his corps to the floor, and put them through a most interesting and instructive drill. It was interesting because it showed perfect control and excellent discipline; it was instructive because it showed the people present just how a hospital corps works in relieving the wounded or sick. Of course, it is not expected that in actual work a squad would go through the work of preparing a stretcher as though they were on exhibition, but this drill showed that by their high proficiency in the exhibition work they would lose no time in emergency work and that there would not be any bungling or confusion.

There are three squads in the 74th's hospital corps and Major Bissell put them through the work of slinging and unslinging stretchers, lifting and returning, and rolling the stretchers. Following this was the rapid preparation of emergency stretchers with rifles and blankets. This was an admirable bit of work and was performed with a precision and rapidity which betokened a high degree of proficiency, of which Major Bissell and the corps should be proud.

Dr. Bissell has not been at the head of the corps many months, but he has brought it up to a high degree of excellence, and Colonel Fox and his regiment should feel proud of the hospital corps, for it is one of the most perfectly drilled as well as one of the best equipped in the National Guard of this state, numbering as it does among its members, druggists, medical students and physicians. The efficiency of the corps was shown first in a most practical way, when, the 65th Regiment sick-train came to Buffalo from Camp Alger, last August, having 109 fever patients. Dr. Bissell and his corps met the train and unloaded the sick. This was no small task, yet it was accomplished tenderly and gently, in less than one-half the time it took to put them on the train at Camp Alger. The 109 patients were removed from the train, properly cared for and disposed of in about forty-five minutes.

Efficiency, intelligence and rapidity characterise the hospital corps of the 74th Regiment, and it well deserves the handsome hospital quarters assigned to it in the new armory.

---

General Leonard Wood, Military Governor of Santiago, who has recently paid a visit to the United States, is one of the most interesting characters that the war with Spain has developed. Being himself a physician by education and practice, until the war, his career has been watched with special interest by physicians. But his many-sided traits, all presenting great points of strength, have made him equally interesting as a splendid specimen of heroic manhood to every American citizen of patriotism and intelligence.

As colonel of the Rough Riders he achieved the first military success in Cuba, and as military governor of Santiago he has set up an object lesson in good government that will prove a difficult ideal for others to attain. He takes a city reeking with the filth that centuries have accumulated, and in a few weeks makes it a model of cleanliness; he falls heir to a death-rate that dirt and tropical diseases have swollen to an alarming size, and reduces it to proportions that even Buffalo may almost envy; he takes a chaotic civil government that is inefficient, if not corrupt, wrecked at all events by war, and rehabilitates it with a matchless speed, making it famous for honesty, justice and economy; he reorganises schools and establishes a comprehensive educational system; he collects the revenues, employs the idle, punishes the criminal, educates the ignorant, and deals kindly with the poor. That the government has recognised his services in part by his recent nomination and prompt confirmation as a major-general of U. S. Volunteers is, indeed, gratifying.

THE New York *Post-Graduate* in a recent issue very appropriately says:

At last we hear the other side. After the daily and medical press have exploited the great mortality from sickness in our war with Spain, we have some facts which we hope our hysterical brethren will consider. The Deputy Surgeon-General of the Army, Lieut. Colonel Charles Smart, states absolutely that the sickness and mortality during the war with Spain were not relatively as great as from that with which our volunteer troops suffered from during the Civil War. Not only, as Dr. Smart says, has time dulled the national memory of what happened in those days, but the volunteer of that time did not expect the sympathy that he seems to have expected in Camps Alger, Chickamauga, Black and Montauk.

After commenting upon Dr. Smart's statistics the *Post-Graduate* continues:

What does this show? It shows that in the five months of the war with Spain, we lost by death 10.21 out of every 1,000 men reported present, and in the first five months of the Civil War we lost 17.31 out of every 1,000 similarly reported present. The medical reports show also that as soon as the general existence of typhoid fever in the camps and of the malarial fever in General Shafter's corps was known, the War Department immediately put forth its energies to remedy this condition, so that the death-rate fell in September to 2.45 as against 4.08 in August. During the Civil War, the death-rate in November, '61, was 4.27, or more than the maximum of August, 1898. In December there was no decrease; the death-rate was 4.69. In January, '62, it rose to 4.84, and in April it was at its height; the death-rate ranged to 8.42 per thousand, twice as high as that reported from our camps in August. This is, indeed, a comparison which ought to produce some remorse in the minds of those journalists and reporters who have filled the air with their outcries of the degeneracy of the United States Army.

---

DURING his recent visit to this country a dinner was given to General Leonard Wood, at the Union League Club, at New York. It was a brilliant affair and among the diners were some of the most distinguished citizens—men of the highest prominence in military, naval, professional and commercial circles. General Wood, of course, made a speech and it is pleasant to observe that, though full of absorbing interest, it was characterised by that same modesty and gentleness, even though bristling with eloquence at times, that has thus far distinguished both his military and civil career. He told of the unfortunate misunderstanding between our people and the Cubans and how it complicated the work of rehabilitation. Listen to his own words as to how the difficulties were in a measure overcome !

There certainly was a good deal of feeling on both sides, and the problem when the old army withdrew and the new one came in was a little difficult, as there was a good deal of sickness and a good deal of distress, in fact more than I have ever dreamed possible in any country, and there was a good deal said of the distress on both sides. But we started in by having no secrets of any kind, shape or description. Everything that was done was done down there as an open book. The secretaries and clerks at department headquarters, in civil departments were all Cubans, and all men who had been in the Cuban army, and all financial transactions were carried out through Cuban clerks, so that everywhere they are now satisfied that as far as the interests of the country went it was all right.

He further told how he dealt with the problem of offices, spoke most interestingly of the respect the inhabitants of eastern Cuba bear toward the American flag and the American people. General Wood concluded his most interesting address as follows :

HEALTH OF THE TROOPS.

The health of the troops may be interesting. When we went there in July, I don't think I ever saw a more pathetic sight than at the departure of our troops as they jumped ashore at Daiquiri and Siboney. We were all inclined at that time to criticise the situation and say that if we had more of something or less of something else it might have been better, but, generally speaking, every man who has gone to Cuba has had fever. Much of the idle talk of defective rations and embalmed beef and other things which none of us have ever seen down there and only heard of after we came home (applause) is not due to the rations, but to the climate. Every soldier going to Cuba is bound to have Cuban fever. The Cubans used to have as high as 60 per cent. and sometimes as high as 80 per cent. unfit for duty. This is simply to show that the condition of our troops was due to the climate, and not the result of any sort of neglect on the part of the government. (Applause.) There are things, of course, that might have been better, but I do not think that any of us expected a picnic.

I want to thank you very much for your more than cordial reception. I cannot half tell you how much I appreciate it. It is this sort of thing that makes one go back with the determination to do the best he can and that is all anyone can do. (Great applause and cheering.)

This should, as it no doubt will, serve to clear the air of much of the fogginess of untruth engendered by the newspapers and medical journals, that have printed material of which they knew little and apparently cared less, if only they could find fault.

IN RELATION to sickness in the Army a recent correspondent of the *New York Tribune* writes to that paper as follows :

Sir—Happening to open an old volume of The Atlantic, I came upon an interesting article on The Sanitary Condition of the Army, which it would be well for all the critics of recent war operations to read. They would discover that sickness is a constant attendant on an army anywhere and under all conditions. One of the most striking cases was in 1809, when the British sent an army of about forty thousand men to the Netherlands, where they were stationed at Walcheren, a place where a previous army, about 1740, had suffered severely from sickness. The second army was attacked by fever and dysentery, like the first, and with similar destructiveness. In two months after landing, 7,626 were on the sick list. In ninety-seven days, more than 12,000 were sent home sick, and in less than four months only 4,000 effective men were left. On the first of the following February there were 11,50c on the sick list, and 15,500 had been lost or disabled. Between January and June of 1810, 36,000 were admitted to the hospital, and 8,000, or more than 20 per cent., died.

And so it goes through every war. If the unreasonable critics of Secretary Alger will turn to the Atlantic Monthly, Volume X., and read the excellent paper by Dr. Edward Jarvis, page 463, it is probable that, while still claiming the very best treatment for our soldiers under all conditions, they will admit that maintaining a large body of men in a campaign fraught with such unusual conditions as existed in the field at Santiago is a most difficult matter, and that while the War Department may deserve censure in some respects, on the whole it deserves from every true American a very large measure of praise.

F. S. D.

---

THE President in his last annual message to the Congress renewed his recommendations of the previous year, relating to the creation of a commission for the purpose of making systematic investigation with reference to the cause and prevention of yellow fever. After introducing the subject the President says :

This matter has acquired an increased importance as a result of the military occupation of the Island of Cuba and the commercial intercourse between this island and the United States, which we have every reason to expect. The sanitary problems connected with our new relations with the Island of Cuba and the acquisition of Porto Rico are no less important than those relating to finance, commerce and administration. It is my earnest desire that these problems may be considered by competent experts and that everything may be done which the most recent advances in sanitary science can offer for the protection of the health of our soldiers in those islands and of our citizens who are exposed to the dangers of infection from the importation of yellow fever. I therefore renew my recommendation that the authority of Congress may be given and a suitable appropriation made to provide for a commission of experts to be appointed for the purpose indicated.

---

THE Grosvenor library has fitted up a special room in its handsome building on Franklin and Edward streets, for the exclusive use of physicians, which it has equipped with 3,500 medical books, and where the current medical magazines will also be found. This room was formally thrown open for the use of physicians on Monday, January 23, 1899, when Mr. Edward P. Van Duzee, the efficient superintendent, and his assistants received the visitors from 1 to 10 p. m.

The room is on the second floor, directly over the entrance on Edward street and is well lighted. It is not intended for the use of students, but of physicians. The students' collection will remain down stairs.

It is one of the best appointed and handsomely prepared rooms for library purposes in the city and ought to be appreciated, as it doubtless will be, by the physicians of Buffalo and vicinity. The trustees of the library, Messrs. Josiah Jewett, E. H. Butler and Ganson Depew, are entitled to the thanks of the medical profession for their liberality in providing such a department of the Grosvenor library, and Mr. VanDuzee, the superintendent, also deserves praise for his efficiency and zeal in so admirably carrying out the wishes of the trustees. The library will be open between the hours of 10 a. m. and 10 p. m. week days and from 1 to 6 p. m. on Sundays. The library will be glad to receive donations of medical books and pamphlets from any person who may have such to spare.

---

SURGEON-GENERAL STERNBERG has been telling what the women did to help on the war to a successful termination. In a letter recently sent to Mrs. J. M. Belden, New York State Regent of the Daughters of the American Revolution, he said :

The want of a sufficient body of trained hospital corps men necessitated the detail of enlisted men from the regiments for hospital duty in several of the camps and the employment of trained nurses at the general hospitals. Foreseeing the necessity for a large force of the latter, I applied to Congress on April 28, 1898, for authority to employ by contract as many nurses as might be required during the war at the rate of $30 per month and a ration, the pay proper to be paid from the appropriation for the medical and hospital department. This was promptly granted. About the same time the National Society of the Daughters of the American Revolution offered its services as an examining board for female nurses, and a committee, of which Dr. Anita Newcomb McGee was chairman, was designated to take charge of the work. Thereafter most of the female nurses employed were selected by this committee, with the exception of those immune to yellow fever who were recruited in New Orleans and other southern cities, and a few who were enrolled at Montauk Point, Long Island, and Jacksonville, Fla., by the chief surgeons at these places.

A number of the patriotic societies offered to provide the hospitals with nurses, but the committee referred to answered its purpose so well that I did not feel the need of additional assistance, and was relieved from what could otherwise have been a serious responsibility.

Over 1,700 female nurses have been employed, at first at the general hospitals and later at the field division hospitals, when it became evident that the field service purposes for which the latter had been organised would have to give place to the imperative need of caring for the many sick men coming from the regimental camps. These hospitals ceased to be ambulance hospitals, and their character of fixed field hospitals was promptly recognised by assigning contract surgeons and nurses to duty with them and providing them with the articles of equipment which cannot be carried in the hospital wagons of a marching command.

Female nurses were not sent to these field hospitals until their original function as an essential adjunct to a command mobilised for active service became lost in the current of immediate necessities. Many of the trained nurses were sisters of charity, whose services were highly appreciated by medical officers in charge as well as by the individual sick men, who benefited by their ministrations. Others were obtained through the kind assistance of the Red Cross Society for the maintenance of trained nurses, Auxiliary No. 3, and I desire to express my high appreciation of the valuable services rendered to the medical department by this organisation.

This will explain the Surgeon-General's real position with reference to employment of trained nurses in the Army, and shows up the falsity of the general belief on the subject created by the misrepresentation of the newspapers.

---

A frequent contributor to the JOURNAL hands us the following letter, which he has recently received:

......................... '98.

Dr................

Buffalo, N. Y.

*Dear Doctor:* We have just read with much interest the paper you presented to the meeting of the . . . , entitled " . . . . . ." Your style of writing is certainly very attractive.

We would like very much to get you to write an article of about a thousand words for our journal. If you will furnish us an article within the next month on one of the following subjects: Micajah's medicated uterine wafers in uterine diseases, manufactured by Micajah & Co., Warren, Pa.; Viskolein, in any disease in which it is indicated, manufactured by the Viskolein Chemical Co., No. 5 Beaver street, New York; Nosophen, in any disease in which it is indicated, manufactured by the Stallman & Fulton, 10 Gold street, New York, we will send you our journal complimentary for three years. Under separate cover we send you several copies of the journal for your inspection. If you need any special literature on the subjects named above, you can get it by writing to the manufacturers, or if you need any of the preparations for experimental use they will take pleasure in sending you all you want. Please let us hear from you by return mail.

Thanking you in advance for a favorable reply, we are,

Very truly yours,

. . . . .

For obvious reasons we suppress names, date, etc., but print the letter to show the extremities to which medical journals are driven, or to which they in many instances voluntarily resort to get or maintain business. And yet the journal whose editor wrote the foregoing ranks as one of the most prosperous provincial medical publications in this country.

